# Oncolytic immunotherapy: unlocking the potential of viruses to help target cancer

**DOI:** 10.1007/s00262-017-2025-8

**Published:** 2017-07-15

**Authors:** Omid Hamid, Brianna Hoffner, Eduard Gasal, Jenny Hong, Richard D. Carvajal

**Affiliations:** 1 The Angeles Clinic and Research Institute, 11818 Wilshire Blvd #200, Los Angeles, CA 90025 USA; 2 University of Colorado Hospital, Aurora, CO USA; 3 Amgen Inc., Thousand Oaks, CA USA; 4 Cedars-Sinai Medical Center, Los Angeles, CA USA; 5 Columbia University Medical Center, New York, NY USA

**Keywords:** Oncolytic immunotherapy, Talimogene laherparepvec, Immunotherapy, Melanoma

## Abstract

Oncolytic immunotherapy is a research area of cancer immunotherapy investigating the use of modified viruses to target cancer cells. A variety of different viral backbones (e.g., adenovirus, reovirus) with a diverse range of genetic modifications are currently being investigated for the treatment of a variety of cancers. The oncolytic virus that has advanced the furthest in clinical development is talimogene laherparepvec, a recombinant HSV-1 virus expressing granulocyte-macrophage colony-stimulating factor (GM-CSF). In a phase 3 study in patients with unresectable metastatic melanoma, intralesional talimogene laherparepvec treatment resulted in a higher durable response rate compared with subcutaneous GM-CSF treatment (16.3 versus 2.1%; *P* < 0.001). Notably, responses were observed at uninjected lesions including visceral lesions, indicating a systemic antitumor response had occurred. Studies evaluating combination treatments involving oncolytic viruses and immunologic agents are ongoing. This review focuses on the mechanisms of action for oncolytic viruses and highlights select agents and combinations currently in development.

## Introduction

**Fig. 1:**
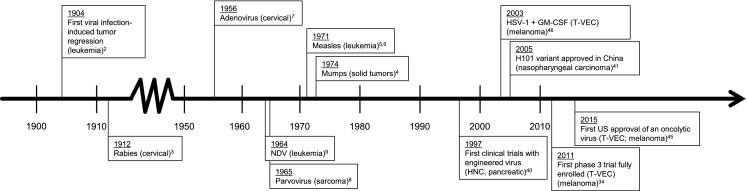
History of oncolytic viruses. *GM*-*CSF* granulocyte-macrophage colony-stimulating factor, *HNC* head and neck cancer, *HSV*-*1* herpes simplex virus type 1, *NDV* Newcastle disease virus, *T*-*VEC* talimogene laherparepvec

Oncolytic immunotherapy is an area of research that investigates the use of modified viruses to induce a systemic immune response to target cancer cells. This represents a novel approach to anticancer therapy; however, the potential for viruses to induce an antitumor immune response has been known for some time. The earliest clinical references to oncolytic viruses were case reports in the early 1900s, primarily describing the remission of malignancies, usually leukemias or lymphomas, after viral infections or live-virus vaccinations [1] (Fig. 1). The first documented case of viral infection–induced regression was in 1904 in a patient with chronic myelogenous leukemia [2]. The patient experienced a marked reduction in white blood cells during a “flu-like” illness. Additional evidence consistent with an ability of viruses to induce an antitumor response was reported over a long period of time. These findings were with a variety of different tumor types (including leukemia, cervical cancer, lymphomas, solid tumors, melanoma, and multiple myeloma) and viruses (including rabies virus, mumps virus, measles virus, adenovirus, parvovirus, and Newcastle disease virus) [1, 3–9]. Together, these observations led to the hypothesis that viruses, and in particular genetically engineered viruses, are immunogenic and might be employed in the treatment of cancer.

**Fig. 2:**
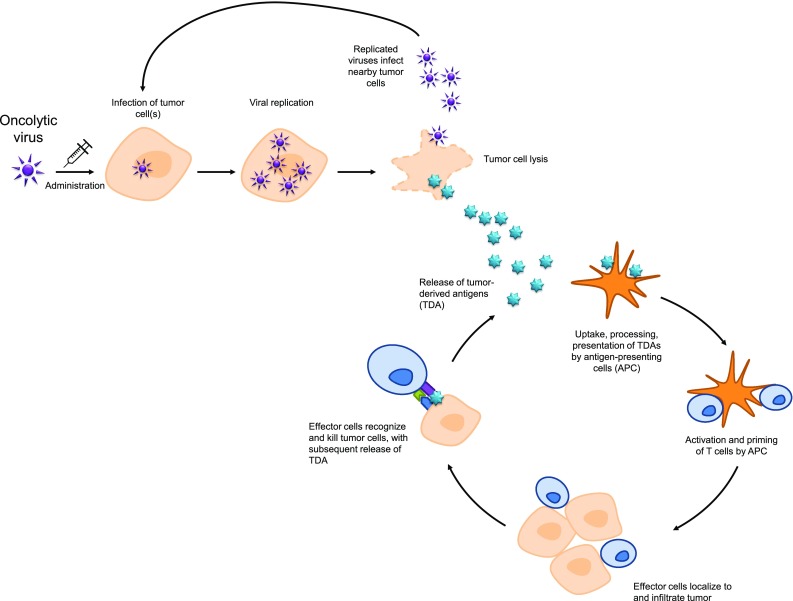
Viral oncolysis mechanism of action and immunogenic response to viral infection. Lysis of tumor cells following viral replication results in release of tumor-derived antigens (TDA), which promote the activity of the cancer-immunity cycle, ultimately resulting in the development of a tumor-specific immune response. *APC* antigen-presenting cells

Oncolytic immunotherapy employs viruses to directly lyse cancer cells (oncolysis). These viruses infect tumor cells, where they undergo a series of replication cycles and are subsequently released through cell lysis to infect adjacent cancer cells. This cycle can repeat hundreds of times, attacking and decreasing the tumor cell mass [1]. Oncolysis also results in the release of tumor-derived antigens that can stimulate a local and systemic antitumor immune response; thus oncolytic therapy can represent an immunotherapeutic approach by which a patient’s own immune system can combat tumor growth and promote tumor removal [1] (Fig. 2). Through the availability of new recombinant DNA technology, oncolytic viruses can be genetically engineered for enhanced activity compared with wild-type viruses. For example, oncolytic viruses can be genetically modified for tumor-selective replication; thus these agents spare neighboring noncancerous tissue while killing targeted cancer cells through lysis. Alternatively, such techniques can also be used to select the most active wild-type viruses for therapeutic use [10].

The goal of this review is to highlight the recently approved oncolytic immunotherapy talimogene laherparepvec, and to describe other oncolytic immunotherapies that are currently in development. The review focuses on the mechanisms of action for engineered oncolytic immunotherapies, important considerations for the clinical development of these agents, current trials under investigation, possible future developments as combinatorial therapies, and the side-effect profiles of oncolytic immunotherapies (which differ from other treatment modalities such as chemotherapy [11]).

## Suggested mechanism of action

Oncolytic viruses can kill cancer cells through a number of mechanisms [12], including (1) direct oncolysis or apoptosis of infected cells [13], (2) apoptotic death of uninfected cells [14], and (3) induction of an immune response [15]. With direct oncolysis, the virus causes lysis or apoptosis of a host cell as a direct result of replication or infection. With replication, the lytic cycle is recapitulated as the viral particles are released and infect neighboring cells [13]. Through this method, the viral load continues to increase until attenuated by an immune response or depletion of susceptible host cells.

Apoptosis is a defense mechanism employed by infected hosts to limit viral spread by eliminating the cellular machinery necessary for viral replication [14]. Oncolytic viruses have been found to be able to trigger apoptosis in neighboring, uninfected cancer tissue [14]. The exact mechanism by which apoptosis is triggered in uninfected cells has yet to be fully elucidated, but theories include transfer of empty virion capsids [16] or binding of viral proteins to extracellular receptors, triggering apoptosis through caspase-mediated mechanisms [17]. Many tumor cells contain defects in the apoptotic pathway (e.g., mutated p53) that allow for tumor growth but usually retain the ability to execute the apoptosis cascade when initiated. Most chemotherapies are unable to trigger this mechanism in p53-deficient tumors because functional p53 is required [18]; however, some oncolytic viruses are designed to circumvent this restriction to trigger apoptosis in cancer cells [18, 19]. Deletion of the viral protein E1, a known inhibitor of apoptosis, allows for an increase in p53 levels leading to apoptosis. Furthermore, some viral proteins are known to induce apoptosis in tumor cells [20]. Certain oncolytic viruses (e.g., vaccinia virus) can also be designed to kill tumor cells by targeting vascular development (angiogenesis), leading to noninfected tumor cell death [21, 22]. In this instance, infection by an oncolytic virus disrupts tumor vasculature, decreasing blood flow to tumor cells, leading to tumor hypoxia [21, 22]. Widespread tumor cell infection and/or necrosis follow these effects at later time points [21].

Tumor cells can also be killed via induction of an immune response [15]. Infected tumor cells are highly immunogenic; the production of cytokines and chemokines and release of tumor-derived antigens from lysed tumor cells can induce a tumor-specific immune response, potentially resulting in the elimination of uninfected cancer cells [15]. Furthermore, a systemic immune response could potentially induce a response at distant uninjected lesions. The potential for oncolytic viruses to induce a tumor-specific immune response has led to the engineering and clinical testing of oncolytic viruses designed to enhance this response, in particular through insertion of genes that encode cytokines [e.g., granulocyte-macrophage colony-stimulating factor (GM-CSF) [23–26], Flt3L [27, 28]] or chemokines (e.g., CCL3 [27], CCL5 [29, 30]).

## Genetic modification of oncolytic viruses

A number of genetically engineered viruses have been created to target specific tissues or tumor types [31] and are being evaluated in clinical trials [32] (Table 1). Engineered viral backbones under clinical investigation include herpes simplex virus type 1 (HSV-1), adenovirus, coxsackievirus A21, reovirus, vaccinia virus, vesiculostomatitis virus, and poliovirus. Recently, the first oncolytic immunotherapy (talimogene laherparepvec; IMLYGIC^™^) was approved by the United States Food and Drug Administration, the European Medicines Agency, and the Australian Therapeutic Goods Administration for the local treatment of unresectable cutaneous, subcutaneous, and nodal lesions in patients with melanoma [33–35].

**Table 1:** Select oncolytic viruses in development

Oncolytic virus	Virus	Transgene(s)	Treatment regimen	Cancer type	Trial identifier(s)^a^	Study phase
DNA viruses						
Talimogene laherparepvec	HSV-1	GM-CSF	Talimogene laherparepvec	Melanoma	NCT00769704	3
			Talimogene laherparepvec	Melanoma	**NCT02297529**	3b
			Talimogene laherparepvec	Melanoma	NCT02147951	3b
			Talimogene laherparepvec	Melanoma	NCT00289016	2
			Talimogene laherparepvec	Melanoma	NCT02366195	2
			Talimogene laherparepvec	Melanoma	**NCT02014441**	2
			Talimogene laherparepvec	Melanoma	**NCT02211131**	2
			Talimogene laherparepvec	Breast	**NCT02658812**	2
			Talimogene laherparepvec + pembrolizumab	Melanoma	**NCT02263508**	1b/3
			Talimogene laherparepvec + pembrolizumab	SCCHN	**NCT02626000**	1b/3
			Talimogene laherparepvec + ipilimumab	Melanoma	NCT02173171	1b/2
			Talimogene laherparepvec + ipilimumab	Melanoma	**NCT01740297**	1b/2
			Talimogene laherparepvec + paclitaxel	Breast	**NCT02779855**	1/2
			Talimogene laherparepvec + radiotherapy	Soft tissue sarcoma	**NCT02453191**	1/2
			Talimogene laherparepvec	Pancreatic	NCT00402025	1
			Talimogene laherparepvec	HCC	**NCT02509507**	1
Seprehvir (HSV1716)	HSV-1	None (ICP34.5 deletion)	HSV1716	Mesothelioma	**NCT01721018**	1/2a
			HSV1716	Solid tumors	**NCT00931931**	1
HF10	HSV-1	None (wild-type)	HF10 + ipilimumab	Melanoma	**NCT02272855**	2
			HF10	HNC, SCC, breast, melanoma	NCT01017185	1
			HF10	Solid tumors	**NCT02428036**	1
ParvOryx (Parvovirus-H1)	Parvovirus-H1	None specified	H-1PV	Glioblastoma	NCT01301430	1/2a
			H-1PV	Pancreatic	**NCT02653313**	1/2
JX-594/PexaVEC	Vaccinia	GM-CSF	PexaVec + sorafenib	HCC	**NCT02562755**	3
			PexaVec	HCC	NCT01387555	2b
			PexaVec	CRC	NCT01394939	1/2a
			PexaVec + cyclophosphamide	Solid tumors, sarcoma, breast	**NCT02630368**	1/2
			PexaVec	Melanoma, lung, RCC, HNSCC	NCT00625456	1
			PexaVec	Pediatric solid tumors	NCT01169584	1
GL-ONC1	Vaccinia	Luciferase-GFP, beta-galactosidase, and beta-glucuronidase	GL-ONC1	Peritoneal	NCT01443260	1/2
			GL-ONC1	Solid tumors	**NCT02714374**	1b
			GL-ONC1	Peritoneal, ovarian, fallopian tube	**NCT02759588**	1b
			GL-ONC1	Lung	**NCT01766739**	1
			GL-ONC1	HNC	NCT01584284	1
			GL-ONC1	Solid tumors	NCT00794131	1
CG0070	Adenovirus	GM-CSF	CG0070	Bladder	**NCT02365818**	3
			CG0070	Bladder	NCT01438112	2/3
			CG0070	Bladder	NCT02143804	2
VCN-01	Adenovirus	PH20 hyaluronidase	VCN-01 + gemcitabine + abraxane	Solid tumors, pancreatic	NCT02045602	1
			VCN-01 + gemcitabine + abraxane	Pancreatic	**NCT02045589**	1
DNX-2401	Adenovirus	None specified	DNX-2401 + pembrolizumab	Brain tumors, glioblastoma	**NCT02798406**	2
			DNX-2401 + IFN-γ	Glioblastoma, gliosarcoma	**NCT02197169**	1b
			DNX-2401 + temozolomide	Glioblastoma	NCT01956734	1
Delta-24-RGD	Adenovirus	E1A deletion	Delta-24-RGD	Glioblastoma	NCT01582516	1/2
Enadenotucirev	Adenovirus	None (wild-type)	Enadenotucirev	Ovarian	**NCT02028117**	1/2
			Enadenotucirev	Solid tumors, mCRC, bladder	**NCT02028442**	1/2
			Enadenotucirev + nivolumab	CRC, bladder, SCCHN, salivary gland	**NCT02636036**	1
ONCOS-102	Adenovirus	GM-CSF	ONCOS-102	Solid tumors	NCT01598129	1
RNA viruses						
MG1MA3	Rhabdovirus	MAGE-A3	MG1MA3	Solid tumors	**NCT02285816**	1/2
VSV-IFN-beta	Vesicular stomatitis virus	IFN-β	VSV-IFN-beta	HCC	NCT01628640	1
Reolysin	Reovirus	None (wild-type)	Reolysin + carboplatin + paclitaxel	SCCHN	NCT01166542	3
			Reolysin + pemetrexed or docetaxel	NSCLC	NCT01708993	2
			Reolysin + docetaxel + prednisone	Prostate	NCT01619813	2
			Reolysin + bevacizumab + FOLFOX	CRC	**NCT01622543**	2
			Reolysin + carboplatin + paclitaxel	Melanoma	NCT00984464	2
			Reolysin + carboplatin + paclitaxel	NSCLC	NCT00861627	2
			Reolysin + paclitaxel	Fallopian tube, ovarian, peritoneal	**NCT01199263**	2
			Reolysin + paclitaxel	Breast	**NCT01656538**	2
			Reolysin + chemotherapy + pembrolizumab	Pancreatic	**NCT02620423**	1b
			Reolysin + chemotherapy + bevacizumab	*KRAS* mutant mCRC	**NCT01274624**	1
			Reolysin + gemcitabine + cisplatin	Bladder	**NCT02723838**	1b
			Reolysin + chemotherapy + bevacizumab	*KRAS* mutant mCRC	**NCT01274624**	1
NDV-HUJ	Newcastle disease virus	None specified	NDV-HUJ	Glioblastoma, sarcoma, neuroblastoma	NCT01174537	1/2
MV-NIS	Measles virus	Human thyroidal sodium iodide symporter	MV-NIS	Myeloma	**NCT02192775**	2
			MV-NIS + gemcitabine + paclitaxel	Ovarian, fallopian, peritoneal	**NCT02364713**	2
			MV-NIS + cyclophosphamide	Myeloma	**NCT00450814**	1/2
			MV-NIS	Ovarian, peritoneal	**NCT02068794**	1/2
			MV-NIS	HNSCC, breast	**NCT01846091**	1
			MV-NIS	Mesothelioma	**NCT01503177**	1
			MV-NIS	Glioblastoma	**NCT00390299**	1
			MV-NIS	Ovarian, peritoneal	NCT00408590	1
			MV-NIS	Peripheral nerve sheath	**NCT02700230**	1
SVV-01	Seneca Valley virus (replication-competent picornavirus)	None specified	SVV-01	Lung	NCT01017601	2
			SVV-01	Neuroendocrine	NCT00314925	1
			SVV-01	Neuroblastoma, sarcoma, kidney	NCT01048892	1
CVA21 (Cavatak)	Coxsackie A21 virus	None	CVA21	Melanoma	NCT01227551	2
			CVA21	Melanoma	NCT01636882	2
			CVA21 + ipilimumab	Melanoma	**NCT02307149**	1b
			CVA21 + pembrolizumab	NSCLC, prostate, melanoma, bladder	**NCT02043665**	1
			CVA21	Melanoma, breast, prostate	NCT00636558	1
			CVA21	Melanoma	NCT00438009	1
			CVA21	HNC	NCT00832559	1
			CVA21 + mitomycin C	Bladder	**NCT02316171**	1
			CVA21 + pembrolizumab	Melanoma	**NCT02565992**	1
PVS-RIPO	Poliovirus Sabin type 1	Human rhinovirus type 2 IRES	PVS-RIPO	Glioblastoma	**NCT01491893**	1
			PVS-RIPO + lomustine	Malignant glioma	**NCT02986178**	2
			PVS-RIPO	Malignant glioma	**NCT03043391**	1

*CNS* central nervous system, *GIST* gastrointestinal stromal tumor, *GM-CSF* granulocyte-macrophage colony-stimulating factor, *HNSCC* head and neck squamous cell carcinoma, *HSV-1* herpes simplex virus type 1, *IFN* interferon, *IL-15* interleukin-15, *IRES* internal ribosome entry site, *MAGE-A3* melanoma antigen family A3, *NSCLC* non–small-cell lung cancer, *SCLC* small-cell lung cancer

^a^Clinical trial data available from www.ClinicalTrials.gov; ongoing trials are in bold

Various approaches have been used to enhance the antitumor activity and tumor selectivity of oncolytic viruses [13, 15]. Modifications may be introduced to influence a number of aspects of the viral mechanism of action. Enhanced tumor selectivity can be achieved by either deleting genes critical for viral replication in healthy cells or by placing tumor-specific promoters of mRNA transcription upstream of those critical genes [13, 32]. Additionally, because mutations in the tumor suppressor genes *p53* and *pRb* result in a loss of cell cycle control in tumor cells, some oncolytic viruses have been engineered to take advantage of this loss of growth control. Engineered viruses containing deletions in genes involved in the inhibition of apoptotic cell death undergo markedly less viral replication in noncancerous cells as compared with tumor cells [13, 32]. For example, an adenovirus with the viral protein E1B55kd deleted (ONYX-015) showed tumor-selective replication that was originally thought to be related to the inability of the mutant protein to degrade p53 [13, 32]. However, more recent data showed that the replication selectivity for tumor cells occurs mainly via an increase in the export and expression of late viral RNAs, which are required for the production of infectious virions [36].

In addition, virulence factors that are important for downregulating the host antiviral response can be deleted to selectively allow replication in tumor cells while sparing normal cells. In the laboratory setting, virulence factor gene deletion has been shown to allow for replication in actively dividing cells (e.g., tumor cells), while reducing proliferation in normal cells [32, 37]. For instance, deletion of the *ICP34.5* gene in HSV-1 attenuated neurovirulence because inhibition of the gene product disables the capacity of the virus to replicate in the central nervous system [38].

Another strategy employs tumor-specific or tissue-specific promoters of transcription to regulate tumor selectivity; the virus is only able to replicate in cells in which the specific promoter is active [13]. Integrating tissue-specific promoters (e.g., osteocalcin promoter) or tumor-specific antigen promoters (e.g., prostate-specific antigen promoter) upstream of viral genes necessary for replication can ensure that proliferation becomes dependent on characteristics unique to tumor cells [32]. The proof of this concept was demonstrated in a 1999 study in which an adenovirus was placed under the control of the α-fetoprotein (AFP) gene promoter and shown to replicate in a hepatocellular carcinoma cell line expressing AFP but not in normal human cells [39].

Lastly, antitumor potency may be enhanced through gene insertion; virus-inducing tumor cell lysis can be augmented by inserting viral genes that encode toxic proteins such as adenoviral death protein or syncytium induction [32]. Furthermore, a local and systemic immune response may be enhanced through the insertion of immune-activating cytokine genes such as human GM-CSF that potentially increase local cytokine expression and tumor-infiltrating lymphocytes. Such genetic modifications can induce inflammation, increase expression of major histocompatibility complex molecules, and activate antigen-presenting dendritic cells, effectively inducing a systemic immune response [32, 40, 41].

## Clinical development considerations for oncolytic immunotherapies

Because introduction of an oncolytic virus elicits an immune response, macrophage infiltration and inflammation can increase tumor dimensions (i.e., pseudoprogression) before clinical improvements are seen [42]. Pseudoprogression has been reported to occur with many immunotherapies, consequently, optimal use of such therapies may require continued therapy past the initial increase in tumor lesion size, with tumor responses observed following initial growth on treatment [11, 23, 24, 43–45]. A study presented at the American Society of Clinical Oncology (ASCO) 2016 Annual Meeting found that in 356 patients with advanced solid tumors treated with anti-PD1, anti-PD-L1, or anti-CTLA-4 antibodies alone or in combination with VEGF or BRAF (V600E) inhibitors, pseudoprogression per immune-related response criteria (irRC) occurred in 21 patients (6%) [46]. Of these 21 patients, 17 (81%) were alive at 1 year [46]. Pseudoprogression has also been reported to occur with oncolytic immunotherapy [47]. Furthermore, even with disease progression following an initial response to therapy, immune responses can be reintroduced with retreatment, effectively eliciting complete and partial responses (PRs) as well as stable disease [32, 48].

Because they have a mechanism of action that differs from other available cancer therapies, oncolytic immunotherapies are anticipated to have potential toxicities that differ from these agents. Adverse events most commonly reported with oncolytic viruses include chills, pyrexia, and influenza-like illness [11, 25, 26, 49, 50]. Because oncolytic immunotherapies employ live viruses, they may cause serious infections in patients who are immunocompromised; therefore patients who are receiving immunosuppressive therapy should not receive additional treatment with oncolytic immunotherapies [33]. Furthermore, careful consideration should be given before administering oncolytic viruses to patients who are receiving antiviral medications as these agents may interfere with the effectiveness of oncolytic therapy [33]. Other potential concerns include natural predisposition toward genomic alterations (i.e., mutations), replication competence (e.g., self-propagation), host-cell selectivity (e.g., tumor specificity), viral shedding, and secondary transmission (e.g., infection of close contacts) [51]. However, these potential concerns have not proven to be of significant concern in clinical studies [52]. No data are available for patients with immune deficiency as clinical trials with oncolytic immunotherapies normally exclude these populations.

The term “viral shedding” means the release of oncolytic or virus-based products from a patient through feces, urine, saliva, other secreted fluids, and through the skin (via sores, wounds, etc.) and describes how a viral product is excreted from a patient’s body [53]. It is a separate and distinct process from biodistribution, which describes how the virus is spread within a patient’s body from the site of administration [53]. The potential for viral shedding has resulted in the development of precautionary administration and handling procedures to limit any risk of secondary transmission of oncolytic viruses [54]. For example, in trials involving the use of talimogene laherparepvec, a modified HSV-1 virus, all healthcare personnel must wear proper personal-protective equipment (e.g., gown, gloves, safety glasses) when handling the virus, in keeping with universal biohazard procedures [53]. The virus is injected into cutaneous, subcutaneous, or nodal lesions with or without ultrasound guidance, and the volume injected is contingent upon the longest lesion size diameter [11]. The maximum volume that could be injected is 4 mL [11, 23]. Once the virus has been administered to the patient, the injection site is disinfected, and after changing gloves, an occlusive dressing is applied. To further minimize the risk of secondary transmission, the outside of the occlusive dressing is disinfected. Materials used for the injection can be disposed of using universal biohazard precautions. If contamination to working surfaces occurs, the contaminated areas are treated with virucidal agents.

## Current oncolytic viruses and trials

Oncolytic viruses have been or are being evaluated as treatment for a variety of malignancies. The first clinical trials using an engineered virus began in the 1990s [55], and a recent search of ClinicalTrials.gov identified approximately 1800 trials involving viruses and cancer. A select number of these are summarized in Table 1, and some representative oncolytic viruses are further elaborated in this section. For example, an engineered H101 variant of adenovirus was approved in China in 2005 for the treatment of nasopharyngeal carcinoma when used in combination with cisplatin [56]. Other viruses under investigation that have recently shown promising results in melanoma include CVA21 (also known as Cavatak), a bioselected variant of coxsackie virus A21, and HF10, a replication-competent oncolytic virus derived from HSV-1 [57, 58]. The most clinically advanced oncolytic immunotherapy is talimogene laherparepvec, a recombinant HSV-1 virus expressing GM-CSF [11, 24] which recently received approval by the United States Food and Drug Administration, the European Medicines Agency, and the Australian Therapeutic Goods Administration for the local treatment of unresectable cutaneous, subcutaneous, and nodal lesions in patients with melanoma.

**Fig. 3:**
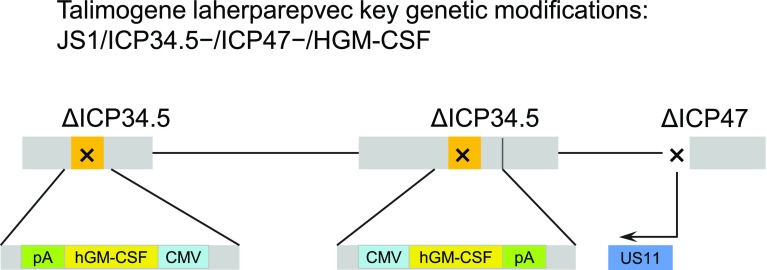
Genetic modifications of talimogene laherparepvec. The viral gene ICP34.5 was deleted and replaced with a human granulocyte-macrophage colony-stimulating factor (hGM-CSF) expression cassette comprising the cytomegalovirus (CMV) promoter, hGM-CSF, and a bovine growth hormone polyadenylation (pA) signal. Expression of the viral gene US11 is driven by the ICP47 promoter

**Fig. 4:**
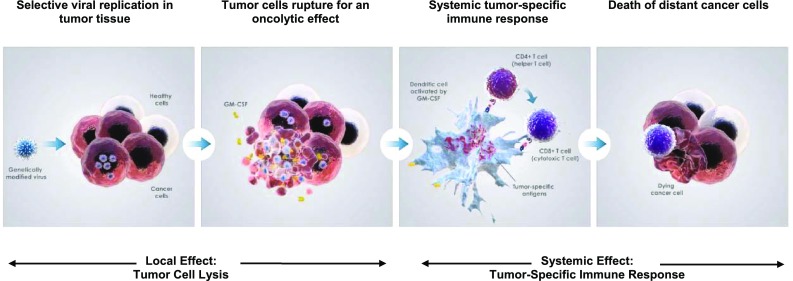
Talimogene laherparepvec proposed mechanism of action. *CMV* cytomegalovirus, *GM*-*CSF* granulocyte-macrophage colony-stimulating factor, *hGM*-*CSF* human GM-CSF, *pA* poly-adenosine, *TDA* tumor-derived antigen

Talimogene laherparepvec is a first-in-class oncolytic virus that has been genetically engineered to selectively replicate within tumor cells when directly injected into lesions and to express GM-CSF to enhance systemic antitumor immune responses. Two viral genes, *ICP34.5* (at two loci) and *ICP47*, were deleted to promote selective replication in tumor cells and to enhance antigen presentation of HSV-infected cells, thus augmenting the immunostimulatory properties of the virus [59] (Fig. 3). The insertion of GM-CSF serves to supplement the antitumor immune response through the recruitment of antigen-presenting cells [60, 61] (Fig. 4).

*ICP34.5*- and *ICP47*-deleted HSV with GM-CSF expression has been shown to increase tumor shrinkage in noninjected tumors and improve the extent of the systemic antitumor response postinjection when compared with the *ICP34.5*- and *ICP47*-deleted HSV without GM-CSF in a syngeneic A20 tumor model [59]. An increase in interferon-γ levels in splenocytes isolated from mice treated with *ICP34.5*- and *ICP47*-deleted HSV expressing GM-CSF was observed when compared with those treated with *ICP34.5*- and *ICP47*-deleted HSV without GM-CSF [59]. Furthermore mice in which tumors were previously cleared by injection with the GM-CSF–expressing mutant HSV were protected against tumor development for 6 months when rechallenged with tumor cells. This implied a systemic antitumor immunity [59]. Clinical data also supported this implication. Results from a phase 2 analysis found an increase in melanoma-associated antigen recognized by T-cells (MART-1)–specific T-cells in tumors from patients treated with talimogene laherparepvec compared with tumors from untreated patients [62]. Moreover, a significant decrease in regulatory T-cells, suppressor T-cells, and myeloid-derived suppressor cells was observed in injected lesions compared with noninjected lesions in the same and different patients [62].

As noted in Table 1, oncolytic viruses are currently being evaluated both as monotherapies and as part of combination therapies in a large number of ongoing trials. Some representative studies are further elaborated in this section [32, 51]. OPTiM, the first randomized phase 3 clinical trial of an oncolytic virus, evaluated durable responses in patients with unresectable stage IIIB/IIIC/IV melanoma (*N* = 436). The primary endpoint was durable response rate (DRR), defined as the rate of complete response (CR) plus PR lasting ≥6 months continuously and beginning within the first 12 months.

**Fig. 5:**
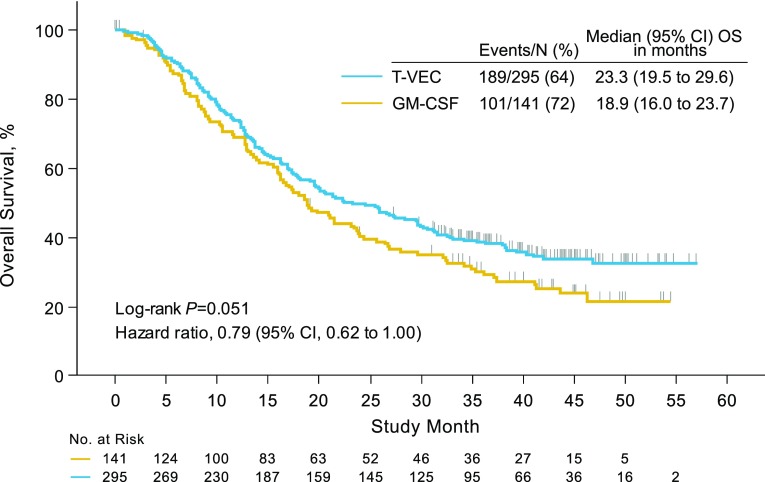
Overall survival after talimogene laherparepvec administration. *GM*-*CSF* granulocyte-macrophage colony-stimulating factor, *OS* overall survival, *T*-*VEC* talimogene laherparepvec. Reprinted with permission from Andtbacka et al. [11]

The study met its primary endpoint. Talimogene laherparepvec resulted in a DRR of 16.3% compared with 2.1% for patients treated with subcutaneous GM-CSF (*P* < 0.001) [11]. The overall response rate (ORR) was 26.4% for patients treated with talimogene laherparepvec compared with 5.7% for patients treated with GM-CSF [11]. Notably, responses (defined as ≥50% decrease in area at a single time point) were observed at uninjected visceral and nonvisceral lesions, indicating that regional and distant systemic antitumor immune responses also occurred. Median overall survival (OS) was 23.3 months with talimogene laherparepvec and 18.9 months with GM-CSF (hazard ratio = 0.79; 95% CI = 0.62–1.00; *P* = 0.051; Fig. 5) [11].

Exploratory subgroup analyses were performed to investigate the treatment effect across key covariates for DRR, ORR, and OS [11]. Differences in DRR between the talimogene laherparepvec and GM‐CSF arms were more pronounced in patients with stage IIIB/C (33 versus 0%) and IVM1a (16 versus 2%) disease than in patients with stage IVM1b (3 versus 4%) and IVM1c (8 versus 3%) disease [11]. Differences in DRR were also more pronounced in patients with treatment‐naive metastatic melanoma (24 versus 0%) than in those receiving treatment as second line or later therapy (10 versus 4%) [11]. A similar pattern was seen for ORR in these subgroups [11]. Treatment effects of talimogene laherparepvec on OS were more pronounced among patients with stage IIIB/C and IVM1a disease (hazard ratio = 0.57; 95% CI = 0.40–0.80; *n* = 249) compared with patients with IVM1b or IVM1c disease (hazard ratio = 1.07; 95% CI = 0.75–1.52; *n* = 186) and among patients with treatment‐naive metastatic melanoma (hazard ratio = 0.50; 95% CI = 0.35–0.73; *n* = 203) compared with those receiving talimogene laherparepvec second‐line or greater therapy (hazard ratio = 1.13; 95% CI = 0.82–1.57; *n* = 233) [11]. Although the reasons for the significant differences in DRR, ORR, and OS observed in patients with stage IIIB/IIIC/IVM1a disease compared with patients with stage IVM1b/IVM1c disease are unknown, these differences might be explained by the talimogene laherparepvec mechanism of action. Disease control in patients with stage IIIB/IIIC or IVM1a disease requires locoregional immune effects, whereas disease control for patients with stage IVM1b or IVM1c disease (i.e., patients with lung or other visceral organ metastases) requires a systemic immune response. It is possible that injection into nonvisceral lesions may activate a systemic immune response that is preferentially directed toward similar metastatic sites rather than to visceral lesions presenting a different antigen pattern [11]. Alternatively, patients with more advanced visceral disease may not have survived long enough to develop systemic antitumor immunity. Differences in overall survival by line of therapy might be explained by the development of immunological defense mechanisms in previously treated tumors, immunosuppressive effects of chemotherapy, or higher baseline tumor burden. Adverse events (AEs; any grade) that occurred most frequently with talimogene laherparepvec treatment included fatigue (50.3%), chills (48.6%), and pyrexia (42.8%) [11]. The most common high-grade AE in the talimogene laherparepvec arm was cellulitis (2.1%) [11].

At the time of the final analysis, conducted 3 years after the last patient was randomized, median (range) follow-up was 49 (37–63) months [63]. Median (95% CI) OS was 23.3 (19.5–29.6) months for patients in the talimogene laherparepvec arm and 18.9 (16.0–23.8) months for patients in the GM-CSF arm (hazard ratio = 0.80; 95% CI = 0.62–1.00; *P* = 0.0494, descriptive) [63]. The 5-year survival rate for patients in the talimogene laherparepvec arm was 33.4% (95% CI = 27.7–39.2) [63].

In a follow-up analysis of the OPTiM trial, 23 of the 48 (48%) patients treated with talimogene laherparepvec who had a durable response experienced progression before response, including 14 patients who developed new lesions only [47]. Pseudoprogression was not found to have a negative impact on survival in this analysis [47].

Further evidence for the activity of oncolytic agents as single-agent therapies is being provided by recent or ongoing investigations. Other oncolytic agents currently being assessed in phase 3 studies include CG0070, a replication-sensitive adenovirus expressing GM-CSF being evaluated in patients with bladder cancer, and pexastimogene devacirepvec (previously, JX-594 or PexaVec), a modified vaccinia virus currently being evaluated in patients with hepatocellular carcinoma (Table 1). In a phase 1 study, the response rate among patients with non–muscle-invasive bladder cancer treated with intravesical infusions of CG0070 every 28 days for three cycles or weekly for six cycles was 48.6% (17/35), and patients treated with the lowest dose [1 × 10^12^ viral particles (vp)] across all treatment schedules had the highest rate of CR (61.5%) compared with other dose levels (range 0–44%) [64]. A median duration of CR of 10.4 months was achieved, with some responses continuing after 17.0 months [64]. Most AEs were grade 1–2, and none were clinically significant. The most common AE observed was dysuria (71.4%) [64]. An open-label, single-arm phase 3 multicenter study of the safety and efficacy of CG0070 in patients with non–muscle-invasive bladder carcinoma who have failed Bacillus Calmette–Guérin therapy and refused cystectomy is currently ongoing (Table 1).

In a small dose-finding study (*n* = 49), treatment of patients with advanced hepatocellular carcinoma with three infusions of intravenous pexastimogene devacirepvec resulted in four objective responses (one CR, three PRs), and ten patients had stable disease [65]. The median OS for all patients was 9.0 months. Median OS was significantly longer for patients in the high-dose group (10^9^ PFU; 14.1 months) compared with the low-dose group (10^8^ PFU; 6.7 months; hazard ratio = 0.39; *P* = 0.02) [65]. Grade 1–2 flu-like symptoms occurred in all patients over the first 12–24 h of treatment; patients in the high-dose group showed a greater temperature increase compared with those in the low-dose group (*P* = 0.002); one grade 4 event of lymphopenia was reported in a patient in the high-dose group [65]. A phase 3 randomized, open-label study comparing pexastimogen devacirepvec followed by sorafenib versus sorafenib in patients with advanced hepatocellular carcinoma without prior systemic therapy is currently ongoing (Table 1).

Initial studies using CVA21 (also known as Cavatak), a bioselected variant of coxsackie virus A21, have been conducted in melanoma, prostate cancer, and breast cancer cell lines and in xenografts in vivo; these cell types all express high levels of receptors (e.g., ICAM-1) that the virus requires for productive infection [66–68]. In all studies, CVA21 infection produced targeted virus-induced oncolysis in both cell culture assays and tumor xenografts [66–68]. In a phase 1 study of patients with stage IV melanoma, five of the nine patients injected with CVA21 had transient/stable reductions in injected tumor volume or tumor stabilization, and two patients had stable disease as defined by Response Evaluation Criteria in Solid Tumors (RECIST) version 1.0 [69]. In a phase 2 study of CVA21 in patients with stage IIIC/IV melanoma (*N* = 57), the immune-related 1-year progression-free survival rate was 28% and the 1-year survival rate was 75% [70]. Initial studies for tolerance of CVA21 are underway in patients with non–muscle-invasive bladder cancer; preliminary results indicate clinical activity and notable signs of viral-induced tumor inflammation [71]. Reolysin^®^, a naturally occurring, unmodified strain of reovirus known for exploiting activated *RAS* signaling, has recently undergone phase 1 and 2 studies in patients with solid tumors [49, 72], and was granted Orphan Drug Designation by the United States Food and Drug Administration in 2015 for the treatment of ovarian cancer [73]. In the most recent study of patients with solid tumors (*N* = 19), treatment was well tolerated in injected patients, and all symptomatic toxicities were mild (grade ≤2) [49]. The most frequently reported AEs were nausea (79%), vomiting (58%), headache (63%), local erythema of injection site (42%), fever and/or chills (37%), dizziness (37%), flu-like symptoms (32%), and diarrhea (32%) [49]. All patients were negative for viral shedding. Best target tumor responses of CR, PR, and stable disease were observed in one patient (5.3%), two patients (10.5%), and four patients (21.1%), respectively [49]. A randomized study of Reolysin^®^ plus chemotherapy in patients with squamous cell carcinoma of the head and neck showed statistically significant improvement in progression-free survival (hazard ratio = 0.536; *P* = 0.007) and OS (hazard ratio = 0.510; *P* = 0.015) compared with chemotherapy alone (ClinicalTrials.gov identifier, NCT01166542); however, it must be noted that this study was not registrational quality [74].

In addition to use as single-agent therapies, oncolytic viruses may have utility when combined with other cancer therapies. In particular, combination of agents (such as oncolytic immunotherapies) that increase tumor-specific immune responses and those that block inhibitory T-cell checkpoints may result in improved antitumor activity compared with treatment with either agent alone. To this end, studies evaluating talimogene laherparepvec in combination with checkpoint inhibitors are currently ongoing. A phase 1b/2 study is assessing combination treatment with talimogene laherparepvec and ipilimumab in patients with melanoma. Results from the phase 1b study (*N* = 19) revealed a confirmed ORR by irRC of 50% [50]. In addition, a disease control rate of 72% (CR, 22%; PR, 28%; stable disease, 22%) was observed with combination treatment [50]. A durable response (CR or PR lasting ≥6 months) occurred in eight patients (44%). Combination therapy with talimogene laherparepvec plus ipilimumab had a median time to response of 5.3 months; initial data indicate disease control within all disease stages [50]. The most common AEs (any grade) were chills, fatigue, and pyrexia (all 58%) [50]. In December 2014, a phase 1b/3, multicenter, open-label trial of talimogene laherparepvec in combination with pembrolizumab for previously untreated, unresected, stage IIIB to IVM1c melanoma was initiated (estimated enrollment, *N* = 660) with the goal of enhancing the antitumor response to either treatment alone [75, 76]. The primary and secondary endpoints of the phase 1b trial include the incidence of dose-limiting toxicities and objective response rate, respectively. Preliminary data from 21 phase 1b patients were presented at the Society for Melanoma Research 2015 Congress [77], and updated results were presented at the 2016 ASCO Annual Meeting [78]. The data suggested that the combination could be administered at full doses with no unanticipated safety concerns. The most common AEs were fatigue (62%), pyrexia (52%), and chills (48%). Efficacy results indicated that among 21 patients who had received their first dose of pembrolizumab at least 12 weeks earlier and had evaluable response assessments, the unconfirmed response rate per investigator was 57%; 24% of patients had an unconfirmed CR. Of these 48% had a confirmed response, and 14% had a confirmed CR [78]. Additional combination studies include the phase 1 STORM (KEYNOTE 200) study, which is evaluating the safety and efficacy of CVA21 in combination with pembrolizumab in patients with non–small-cell lung cancer and bladder cancer [79] and PHOCUS, the randomized phase 3 study of pexastimogene devacirepvec, followed by sorafenib versus sorafenib alone in patients with hepatocellular carcinoma [80]. Both studies are currently recruiting participants, with results expected in 2019 and 2017, respectively.

Oncolytic therapy has also been studied in combination with chemotherapy and radiation treatments [56, 81–87]. Combination treatment with ONYX-015, cisplatin, and 5-fluorouracil resulted in tumor shrinkage in 25 of 30 cases of head and neck cancer. The ORR for the intent-to-treat population (*N* = 37) was 53%; 8 (27%) had CR and 11 (36%) had a PR [83]. Treatment was well tolerated, and a lack of cross-resistance was seen between chemotherapeutic and virotherapeutic agents [83]. Another trial evaluated an adenovirus containing two suicide genes, *CD* and *HSV*-*1 TK*, which convert the prodrug 5-fluorocytosine to 5-fluorouracil and thymidine analogs to their monophosphate analogs, respectively, effectively sensitizing infected cells to ionizing radiation. In a trial of patients with prostate cancer (*N* = 15) who were treated with the adenovirus, the two prodrugs, and radiation therapy, all patients saw significant declines in prostate antigen levels after combination treatment [81]. Lastly, a phase 1 study of ONCOS-102, an adenovirus expressing GM-CSF in combination with cyclophosphamide, was recently completed in patients (*n* = 10) with solid tumors [88]. No dose-limiting toxicities occurred and four patients had disease control at 3 months; median OS was 9.3 months [88].

## The future of oncolytic immunotherapy

Although the theoretical promise of oncolytic immunotherapy has been demonstrated, it is likely that additional gains will require combinatorial treatment with other therapeutic modalities. For example, combination treatment with both immunotherapeutic agents and chemotherapeutic regimens has been evaluated with a number of viral backbones (e.g., ONYX-015, H101, talimogene laherparepvec) [1, 50, 56]. As mentioned previously, talimogene laherparepvec is currently being evaluated in combination with ipilimumab or pembrolizumab in patients with melanoma, and it is likely that other combinations with checkpoint inhibitors will be investigated in the future.

Furthermore, through genetic modification, there are additional opportunities to engineer oncolytic viruses that specifically deliver different payloads to tumor targets. For example, one study evaluated the antitumor efficacy of a transductionally and transcriptionally targeted oncolytic adenovirus that was armed with a fully human monoclonal antibody targeted toward CTLA4-Ipilimumab (Ad5/3-ΔaCTLA4) [89]. Treatment with the virus was able to activate T-cells from cancer patients, as measured by increases in interleukin-2 and interferon-γ levels [89]. In addition, a direct apoptotic effect was observed with viral treatment, both in vitro and in vivo, and the apoptotic effect directly correlated with anti-CTLA4 antibody expression with phase 1b response rates over 50% [89]. Another example is a genetically engineered rhabdovirus Maraba (MG1) expressing a melanoma-associated tumor antigen, which has been evaluated in preclinical study in mice [90]. Alone, the MG1 vaccine does not appear to be sufficient to invoke adaptive immunity against the antigen, but when tested with a heterologous prime-boosting vector (recombinant adenovirus expressing human dopachrome tautomerase), MG1 quickly generated strong, antigen-specific T-cell responses and prolonged survival [90].

## Conclusion

Oncolytic immunotherapy is a multifaceted and promising treatment option in the field of cancer immunotherapy. The quantity and variety of virus types, genetic modifications introduced, and therapy combinations being evaluated in preclinical and clinical studies are numerous and continuing to grow. It will be interesting to see the progress and potential of these exciting therapeutic strategies in future clinical trials.

## Abbreviations

AE: Adverse event
AFP: α-Fetoprotein
CR: Complete response
DRR: Durable response rate
GM-CSF: Granulocyte-macrophage colony-stimulating factor
HSV-1: Herpes simplex virus type 1
MART-1: Melanoma associated antigen recognized by T-cells
ORR: Overall response rate
OS: Overall survival
PR: Partial response
